# Recent Advances in Understanding the Structural and Functional Evolution of FtsH Proteases

**DOI:** 10.3389/fpls.2022.837528

**Published:** 2022-04-06

**Authors:** Lanbo Yi, Bin Liu, Peter J. Nixon, Jianfeng Yu, Feng Chen

**Affiliations:** ^1^Institute for Food and Bioresource Engineering, College of Engineering, Peking University, Beijing, China; ^2^Shenzhen Key Laboratory of Marine Microbiome Engineering, Institute for Advanced Study, Shenzhen University, Shenzhen, China; ^3^Institute for Innovative Development of Food Industry, Shenzhen University, Shenzhen, China; ^4^Sir Ernst Chain Building-Wolfson Laboratories, Department of Life Sciences, Imperial College London, London, United Kingdom

**Keywords:** FtsH protease, mitochondria, protein engineering, functional adaptation, evolution, chloroplasts, photosystem II repair

## Abstract

The FtsH family of proteases are membrane-anchored, ATP-dependent, zinc metalloproteases. They are universally present in prokaryotes and the mitochondria and chloroplasts of eukaryotic cells. Most bacteria bear a single *ftsH* gene that produces hexameric homocomplexes with diverse house-keeping roles. However, in mitochondria, chloroplasts and cyanobacteria, multiple FtsH homologs form homo- and heterocomplexes with specialized functions in maintaining photosynthesis and respiration. The diversification of FtsH homologs combined with selective pairing of FtsH isomers is a versatile strategy to enable functional adaptation. In this article we summarize recent progress in understanding the evolution, structure and function of FtsH proteases with a focus on the role of FtsH in photosynthesis and respiration.

## The Basics of FtsH Proteases

### Discovery of FtsH

FtsH proteases belong to the ATPase Associated with diverse cellular Activities (AAA+) super family. FtsH was first identified by Santos and De Almeida following the isolation of a mutant of *Escherichia coli* that displayed elongated cells and was sensitive to heat-shock, and hence named *filamentous temperature sensitive H* (*ftsH*) (Santos and De Almeida, 1975). However, later studies demonstrated that the *ftsH* mutation was only responsible for heat-induced growth arrest and not the filamentation defect (Ogura et al., 1991). FtsH orthologs were later identified in almost all cellular organisms except for some archaebacteria (Summer et al., 2006; Wagner et al., 2012; Giménez et al., 2015). For a period of time, *E. coli* FtsH was also designated as HfIB until it was confirmed to be encoded by the same gene (Herman et al., 1993). FtsH proteases have been found to target a broad-range of proteins, both membrane-bound and soluble, and to participate in the regulation of diverse pathways (Ogura et al., 1999; Ito and Akiyama, 2005; Janska et al., 2013; Kato and Sakamoto, 2018).

### The Layout of Domains in the FtsH Primary Structure

**FIGURE 1:**
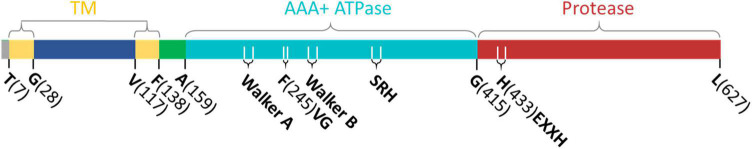
Schematic representation of domains of FtsH2 from *Synechocystis* sp. PCC 6803. Annotations: transmembrane domain (TM), ATPase domain (AAA + ATPase) and protease domain (Protease) are illustrated as colored boxes. The TM domain includes two transmembrane helices (yellow) and a soluble linker exposed to the thylakoid lumen (blue). The soluble regions exposed to the cytoplasm include a flexible linker (green), ATPase (cyan), and protease domains (red). The amino-acid residues that separate each domain are labeled below the colored boxes. Annotated functional motifs include the proposed substrate entry point (FVG); ATP hydrolysis motifs Walker A and B, and the second region of homology (SRH); and the zinc-binding proteolytic site (HEXXH, X represents any amino acid).

FtsH proteases are present in prokaryotes, and mitochondria and chloroplasts of eukaryotic cells (Adam et al., 2005; Tatsuta and Langer, 2009). The typical bioinformatic features of FtsH (Figure 1) include an N-terminal transmembrane domain consisting of one or two transmembrane helices, followed by a highly conserved AAA+ ATPase domain containing Walker A and B structural elements, a second region of homology motif that is responsible for the binding and hydrolysis of ATP (Ito and Akiyama, 2005; Suno et al., 2006), and a downstream M41 peptidase domain featuring a zinc-binding proteolytic site capable of digesting unfolded proteins into ∼12 amino-acid long oligopeptides (Suno et al., 2006). The ATPase domain is highly conserved across species, while the C-terminal tail of the protease domain and the regions connecting the transmembrane domains are less conserved (Langer, 2000; Suno et al., 2006; Graef et al., 2007).

### Formation of FtsH Complexes

**FIGURE 2:**
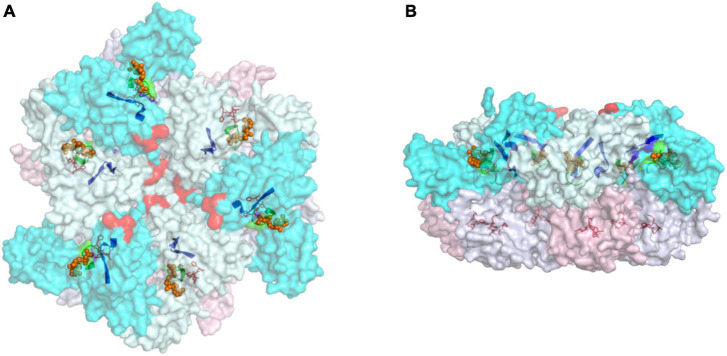
Top **(A)** and side **(B)** views of the cytoplasmic region of hexameric FtsH complex from *T. thermophilus* (PDB:2DHR). Annotations: protomers are defined according to the status of its ATPase domains (“open” confirmation in cyan and “closed” confirmation in pale-cyan). The protease domains of “open” protomers are labeled pink, and the “closed” counterparts are in blue-white. Selected motifs presented in a non-translucent color include Walker A (green ribbon) and B (blue ribbon), and the zinc coordinates of the protease site (red sticks). The proposed FVG motif responsible for substrate binding is displayed as a red sphere, and ADP molecules are shown as orange spheres.

Both *in vivo* and *in vitro* studies suggest that the proteolytic activity of FtsH requires the formation of oligomeric complexes (Akiyama et al., 1995; Krzywda et al., 2002; Suno et al., 2006; Lee et al., 2011; Boehm et al., 2012). Structural data reveal that an FtsH complex consists of six protomers, with the soluble ATPase and protease domains interacting with the neighboring protomers to form a hexagonal particle (Figure 2; Suno et al., 2006; Lee et al., 2011; Boehm et al., 2012). In organisms containing multiple FtsH homologs, the pairing of protomers is strictly regulated, resulting in the formation of distinctive FtsH homo- and heterocomplexes conferring specialized functions (Lee et al., 2011; Boehm et al., 2012; Gerdes et al., 2012; Wagner et al., 2012; Scharfenberg et al., 2015; Kato and Sakamoto, 2018). In most cases, FtsH homologs form either homohexamers or heterohexamers. Exceptions include Afg312, a human mitochondrial FtsH, that can form both homocomplexes and heterocomplexes with paraplegin (Casari et al., 1998; Karlberg et al., 2009; Gerdes et al., 2012; Patron et al., 2018) and FtsH3 and FtsH10 in *Arabidopsis* mitochondria which form both homo- and heterocomplexes (Piechota et al., 2010). A variety of experimental data suggest that FtsH heterocomplexes in diverse organisms contain two types of FtsH protomer in a 1:1 stoichiometry (Lee et al., 2011; Boehm et al., 2012; Langklotz et al., 2012; Kato and Sakamoto, 2018; Steele and Glynn, 2019). However, possible exceptions are some type A/type B FtsH heterocomplexes isolated from *Arabidopsis* which have been suggested to contain the two forms in a 1:2 ratio (Moldavski et al., 2012) although a recent study supports a 1:1 ratio (Kato et al., 2018).

Studies on FtsH heterocomplexes show that the pairing of FtsH isomers is highly specific, hence the formation of hexamers requires a selective matching mechanism. Early research suggested that the transmembrane domain helps homo-oligomerization of *E. coli* FtsH (Makino et al., 1999). However, later studies on FtsH from *Thermophilus maritima* and *Thermus thermophilus* demonstrated that heterologous expression of the ATPase domain, protease domain, or the complete cytosolic domain, can still form hexamers (Langklotz et al., 2012). Therefore, the tendency to form hexameric oligomers is determined by the soluble domains. Mutations in the protease domain have been shown to convert yeast Yta10/Yta12 FtsH heterocomplexes into Yta12 homocomplexes (Lee et al., 2011). This work implies that the structure of the protease domain plays a key role in selecting the appropriate FtsH protomer. However, the residues responsible for determining specificity are not totally conserved in FtsH sequences, and the resolution of the yeast FtsH structure is still too low to identify specific interactions for these residues in the complex. Therefore, the underlying mechanism requires further investigation.

### The Molecular Mechanism of FtsH Complexes

It is widely accepted that FtsH complexes conduct ATP-dependent proteolysis by unfolding and translocating the target substrate through the central pore of the ATPase complex to the protease domain for degradation (Yamada-Inagawa et al., 2003; Okuno et al., 2006; Suno et al., 2006; Carvalho et al., 2021). However, the detailed molecular mechanism for substrate recognition and proteolysis is still under investigation. Current models suggest a ∼20 amino acid flexible linker between the transmembrane and ATPase domains creates space for substrates to gain access to the protease (Lee et al., 2011; Puchades et al., 2017; Carvalho et al., 2021). A phenylalanine residue positioned on the top surface near the central pore of ATPase domain (in the FVG motif) is responsible for substrate binding (Bieniossek et al., 2006; Narberhaus et al., 2009). ATP hydrolysis induces conformational changes in the ATPase domain so that the phenylalanine slides into the central pore with the substrate (Suno et al., 2006). For each ATP-driven movement, the substrate can be translocated ∼35 Å in distance with the unfolded peptide chain translocated via internal channels to the protease catalytic site for degradation. From an energy perspective, 8 ATP molecules are consumed for each peptide cleavage reaction based on experimental data (Murata and Nishiyama, 2018). Theoretical considerations suggest 6 ATP molecules per cleavage event is the minimum requirement (Suno et al., 2006).

Multiple atomic-resolution structures of FtsH, mostly of the soluble domains of homocomplexes from bacteria, are available in the protein databank (Bieniossek et al., 2006; Suno et al., 2006; Puchades et al., 2017; Carvalho et al., 2021). The structures reveal that the protease domains form a rigid hexameric “disk” underneath the ATPase domains; whereas the ATPase “disk” is flexible (Bieniossek et al., 2006; Suno et al., 2006). The structural symmetry of the ATPase domains is inconsistent across species. Early works on *E. coli* FtsH (PDB: 1LV7) suggested a sixfold symmetry for the ATPase domains (Krzywda et al., 2002; Scharfenberg et al., 2015). FtsH structures from *Aquifex aeolicus* (PDB: 4WW0) and *Thermotoga maritima* (PDB: 2CE7 and 2CEA) suggest that the ATPase domains display a twofold symmetry in the ADP-bound state (Bieniossek et al., 2006, 2009; Vostrukhina et al., 2015). Two protomers on the opposite side of the central pore display an identical interdomain angle that is different to the other four protomers. In a separate study, the crystal structure of an ADP-bound FtsH complex from *T. thermophilus* (PDB:2DHR) revealed a threefold symmetry (Suno et al., 2006) so that the ATPase domain displays alternating “open” or “closed” conformations in the ring. In contrast, analysis of a cryo-EM structure of the yeast mitochondrial Yme1 mitochondrial homocomplex has revealed a spiral staircase conformation of the ATPase domains which has led to the suggestion that ATP hydrolysis leads to progressive rotary conformational changes (Puchades et al., 2017).

We speculate that the differences in symmetry among FtsH structures might be artifacts of crystallization and cryo-EM, as the sequence identity and structure of the ATPase domains are highly conserved and hence likely to operate in a similar fashion. In addition, the “staircase” structure of Yme1 was obtained from an ATPase-inactivated mutant. On the flipside, it is plausible that FtsH orthologs have adopted different ATP hydrolysis strategies during evolution to improve energy efficiency. Depending on the nature of the substrates and reaction conditions, energy-dependent enzymatic activities require optimization of power and efficiency. For example, the number of c-subunits of ATP synthases in each species is adjusted to achieve a specific proton per ATP ratio for balanced performance (Kramer and Evans, 2011).

The gap between the ATPase domain and membrane is considered a limiting factor for substrates to gain access to the protease (Lee et al., 2011; Carvalho et al., 2021). Structural studies on native FtsH from yeast determined the gap to be ∼13 Å, which would suggest that only unfolded substrates can reach the central pore (Lee et al., 2011). However, recent structural and mutagenesis work suggests that the gap can be enlarged via tilting of the linker region (Carvalho et al., 2021). Therefore, the width of the gap may not be a strict gatekeeper element for substrate specificity.

### FtsH Supercomplexes

*In vivo* FtsH activities are regulated by members of the band 7 (or SPFH) protein family that includes the stomatins, prohibitins, flotillins and HflK/C (Tavernarakis et al., 1999; Gehl and Sweetlove, 2014). In *E. coli*, FtsH activity is negatively regulated through the action of a large complex consisting of the HflK and HflC subunits (Kihara et al., 1996, 1997, 1998; Akiyama, 2009). A recent cryo-EM structure of a HflK/C-FtsH super-complex has revealed that the HflK and HflC subunits assemble within the membrane to form a ring with a large dome in a “hat-like” structure (Ma et al., 2022). Up to four FtsH complexes can be contained within the HflK/C complex. The transmembrane helices of HlfK and HflC form a circular barrier that prevents the FtsH complexes from moving out into the surrounding membrane thereby potentially preventing unwanted degradation of proteins. The work also showed that FtsH activity is negatively correlated with the abundance of HflK/C. The related prohibitin complexes might play a similar role in regulating FtsH activity in mitochondria (Steglich et al., 1999) and cyanobacteria (Boehm et al., 2012; Bečková et al., 2017; Zhang et al., 2021), and more generally members of the SPFH family could form ring-like structures in the membrane and have scaffolding functions (Gehl and Sweetlove, 2014; Wai et al., 2016).

### The Cellular Locations and Functions of FtsH Proteases

In *E. coli*, there are five proteases (ClpXP, ClpAP, HslUV, Lon and FtsH) of the AAA+ protease family involved in ATP-dependent proteolysis (Langklotz et al., 2012). Of these, FtsH and Lon proteases are membrane-anchored, and FtsH is essential for cell viability (Ogura et al., 1999). In most prokaryotes, FtsH is encoded by a single gene, and localized in the cytoplasmic membrane (Tomoyasu et al., 1995). In cyanobacteria, typically 4 FtsH homologs are present which form specialized complexes in the cytoplasmic and thylakoid membranes (Boehm et al., 2012; Sacharz et al., 2015). In eukaryotes, FtsHs are exclusive to mitochondria and chloroplasts in accordance with the prokaryotic origin of FtsH proteases (Wagner et al., 2012). However, all FtsH homologs in eukaryotes are encoded by nuclear genes, implicating gene transfer during endosymbiosis.

FtsH proteases perform both protease and chaperone roles to maintain cellular homeostasis (Wagner et al., 2012). Previous studies revealed that FtsH in *E. coli* cells regulates heat-stress response via degradation of heat-shock transcription factor σ^32^ (Tomoyasu et al., 1995). FtsH is also required to balance the turnover rate of LpxC deacetylase, an important regulatory mechanism to maintain the lipopolysaccharide/phospholipid ratio (Ogura et al., 1999). FtsH plays a crucial role in removing inactive membrane-bound Sec translocons (Kihara et al., 1995; Akiyama et al., 1996). Interestingly, rapid removal of Sec translocons by FtsH can be a greater risk to cell viability than allowing inactivated Sec translocons to accumulate (Kihara et al., 1995). Chaperone activities of FtsH have been reported from studies on bacteria, mitochondria and chloroplasts (Ogura et al., 1991; Bailey et al., 2001; Wagner et al., 2012; Li et al., 2013; Mishra et al., 2019). FtsH is essential in *E. coli*, *Bradyrhizobium japonicum*, *Helicobacter pylori* and *Borrelia burgdorferi* (Wang et al., 2021); whereas, it is dispensable in *Bacillus subtilis*, *Lactococcus lactis*, *Caulobacter crescentus*, *Staphylococcus aureus*, and *Pseudomonas aeruginosa* (Kamal et al., 2019). However, the lack of FtsH reduces their cell fitness (Chu et al., 2016).

FtsH proteases are safeguards of photosynthesis and respiration and are found in the thylakoid and the mitochondrial inner membranes, where the major photosynthetic and respiratory electron transport machineries are located. Both photosynthesis and respiration are subject to oxidative stress, hence the proteins involved are often short-lived due to damage by reactive oxygen species (ROS) (Krynická et al., 2014; Steele and Glynn, 2019). Spatially coordinated FtsH complexes can efficiently recognize and remove damaged proteins, thus leave room for *de novo* produced replacements to be incorporated to reactivate the pathway (Janska et al., 2010; Nixon et al., 2010; Kato and Sakamoto, 2018). In the case of photosynthesis, efficient FtsH-mediated repair of damaged photosystem II (PSII) is an important determinant of primary productivity (Nixon et al., 2005; Boehm et al., 2012; Wagner et al., 2012).

## Photosynthesis and FtsH

### Photosynthesis and Photosystem II Repair

Oxygenic photosynthesis is the primary energy harnessing process on Earth and a prominent source of oxygen for the atmosphere (Nelson and Ben-Shem, 2004). Water photolysis catalyzed by PSII generates molecular oxygen and reducing power to produce organic matter. The process is chemically challenging due to the high redox potentials generated in the reaction center, which leads to protein damage (Vass et al., 2007; Vass, 2012; Bricker et al., 2015). Nature’s solution to mitigate photodamage is to restrict hazardous reactions to a single subunit, namely the D1 protein, to enable rapid replacement of inactivated D1. The PSII repair strategy is energy efficient as it enables recycling of nearly all the subunits and cofactors of PSII (Nixon et al., 2010; Nickelsen and Rengstl, 2013).

Maintaining photosynthetic activity requires the PSII repair rate to match the rate of damage to D1. When D1 damage exceeds repair, inactivated PSII accumulates, and net photosynthetic rate falls. This phenomenon, termed chronic photoinhibition, can be lethal if prolonged (Aro et al., 1993; Edelman and Mattoo, 2008; Nixon et al., 2010; Nickelsen and Rengstl, 2013). Removal of damaged D1 is a crucial step that leads to reassembly and reactivation of PSII. The current view is that upon photodamage, PSII undergoes partial disassembly which allows D1 to be exposed for replacement (Krynicka et al., 2015). Specialized FtsH complexes extract D1 from the membrane which allows the replacement copy to be assembled into the PSII chassis (Bailey et al., 2001; Silva et al., 2003; Nixon et al., 2005).

Efficient D1 replacement is vital to maintain photosynthetic activity. D1 is the most conserved protein known to date (Cardona et al., 2019; Sánchez-Baracaldo and Cardona, 2020), and consists of 5 transmembrane helices with a peripheral N-terminal tail that is crucial to initiate degradation (Komenda et al., 2007), and a C-terminal post-translational cleavage site for controlled assembly of the inorganic oxygen-evolving complex (Nixon et al., 1992; Anbudurai et al., 1994; Oelmüller et al., 1996; Suorsa and Aro, 2007). Under strong illumination, D1 is among the fastest turned-over proteins in cells, with a half-life as short as 15–20 min (Jansen et al., 1996, 1999; Yao et al., 2012; Kale et al., 2017). It is reported that the biosynthesis rate of D1 in mature chloroplasts accounts for 50% of net protein synthesis, however, the abundance of D1 is only ∼0.1% of protein content (Nelson and Ben-Shem, 2004). Hence the rate and efficiency of D1 replacement are important metrics to evaluate the productivity and light tolerance of a photoautotroph.

### Role of FtsH in Photosystem II Repair

Thylakoid FtsH has been shown to selectively remove damaged D1 in both cyanobacterial and plant models. Initial suggestions for a role for FtsH came in 1999, when Spetea et al. (1999) reported that D1 degradation was a multi-stage, ATP- and zinc-dependent process using an *in vitro* proteolysis assay; and suggested multiple proteases including thylakoid FtsH could be involved. Since then, extensive mutagenesis works have been conducted to evaluate the significance of each protease candidate (Bailey et al., 2002; Silva et al., 2003; Huesgen et al., 2006; Kapri-Pardes et al., 2007). In both plants and cyanobacteria, only FtsH complexes are essential for efficient PSII repair (Nixon et al., 2005).

**FIGURE 3:**
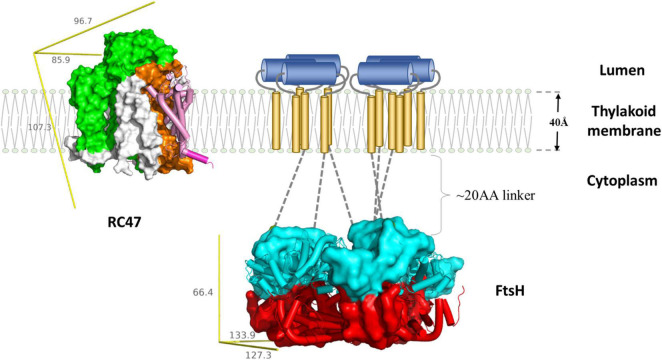
Schematic representation of FtsH-mediated D1 degradation. Upon inactivation, PSII undergo partial disassembly, resulting in transient complex RC47 (PDB:4V62) containing inactivated D1 (pink and purple), D2 (orange), CP47 (green), and small chlorophyll-binding subunits (white). The N-terminal loop of D1 (purple cylinder) is exposed for degradation. The thylakoid FtsH complex (adapted using crystal structure of the whole cytosolic domains of FtsH from *T. thermophilus*, PDB:2DHR) participates in D1 removal. In the current model, the N-terminal D1 loop feeds through the FtsH linker region (gray dotted lines) and is pulled by the ATPase domains (cyan) down the central pore, and translocated to the protease domain (red). Both RC47 and FtsH complexes are plotted to scale using the lipid membrane as size reference. The missing transmembrane region of FtsH is illustrated as a cartoon: transmembrane helices (gold cylinders) and lumenal region (blue cylinders). The yellow reference rulers represent the dimension (unit: Å) of the Inertia Axis Aligned Bounding Box (IAAB) for the respective protein, which are calculated by Guardado-Calvo’s Python scripts for pymol (https://pymolwiki.org/index.php/Draw_Protein_Dimensions).

The molecular mechanism of FtsH-mediated D1 degradation remains elusive. Pulse-chase labeling experiments show that newly synthesized D1 accumulates as an unassembled membrane protein in an FtsH2-deficient *Synechocystis* mutant, indicating FtsH-mediated proteolysis of damaged D1 is vital for D1 incorporation (Silva et al., 2003; Komenda et al., 2006). FtsH complexes co-purify with PSII complexes (Silva et al., 2003) but the direct interaction between FtsH and D1 has yet to be observed, possibly due to rapid D1 degradation. D1 mutants lacking 20 amino-acids of the N-terminal tail display an FtsH null-like phenotype in cyanobacteria (Komenda et al., 2007) which would suggest that the N-terminal tail of D1 exposed on the membrane surface is crucial to initiate proteolysis. Hence in the current D1 degradation model (Figure 3), the N-terminus of D1 facilitates the initial contact with the FVG motif of an FtsH complex and is then pulled into the central pore of FtsH for degradation. FtsH complexes involved in PSII repair are heterohexameric and composed of type A and type B FtsH subunits (Sakamoto et al., 2003; Boehm et al., 2012). Site-directed mutagenesis of type B FtsH protomers has revealed that inactivation of ATPase activity compromises PSII repair, but that inactivation of the protease activity does not (Zhang et al., 2010; Yu, 2013). Hence only one functional protease domain might be needed for FtsH function. The hypothesis is also in agreement with the structural model proposed from a *T. thermophilus* FtsH study (Suno et al., 2006).

Cells lacking thylakoid FtsH can maintain photosynthesis under low light intensity. Photodamage is inevitable even under low irradiance, which indicates the presence of auxiliary D1 degradation routes (Silva et al., 2003; Adam et al., 2005; Nixon et al., 2005). Increased accumulation of truncated D1 fragments is observed in the *Arabidopsis* and *Chlamydomonas reinhardtii* FtsH mutants, highlighting the involvement of other proteases, although cleavage by ROS cannot be ruled out (Kato et al., 2012; Malnoë et al., 2014).

*In vitro* biochemical analyses indicate Deg proteases, a class of ATP-independent serine proteases, as responsible for the cleavage of D1 in the lumenal loop region between transmembrane helix C and D (Sun et al., 2007; Kato et al., 2012). 16 Deg proteases are found in *Arabidopsis thaliana*, of which Deg2 and Deg7 are localized in the stroma (Sun et al., 2010; Luciński et al., 2011); whereas, Deg1, Deg5 and Deg8 reside in the thylakoid lumen (Kapri-Pardes et al., 2007; Sun et al., 2007). The potential benefit from Deg cleavage is that once D1 is broken into multiple smaller degradants, additional proteases, such as Clp can be involved to accelerate D1 replacement (Kato et al., 2012). Several studies on *Arabidopsis* mutants lacking Deg protease subunits observed reduced D1 repair rate under strong illumination, hence the involvement of Deg proteases in D1 repair is physiologically relevant, too (Kapri-Pardes et al., 2007; Sun et al., 2007, 2010; Schuhmann and Adamska, 2012). However, an *Arabidopsis* mutant lacking Deg1, 5, and 8 is still able to grow photoautotrophically unless challenged with strong abiotic stress (Butenko et al., 2018). In contrast, mutants lacking all type A or type B FtsH subunits abolish autotrophic growth and are embryo lethal (Yu et al., 2004, 2005; Zaltsman et al., 2005a). Therefore, Deg proteases play a more minor role in PSII repair (Yu et al., 2004, 2005; Zaltsman et al., 2005a).

However, in the *Synechocystis* model, mutants lacking all three annotated Deg proteases do not display measurable defects in PSII repair, which casts doubt over the universality of Deg-mediated cleavage (Barker et al., 2006). It is possible that D1 turnover in viridiplantae is more sophisticated as they possess more complex thylakoid structures and are typically exposed to stronger light irradiance. Overall, current models suggest that FtsH complexes are the main proteases involved in D1 degradation in both cyanobacteria (Silva et al., 2003) and chloroplasts (Kato et al., 2009) and that the Deg and Clp proteases play a supplementary role (Kato and Sakamoto, 2018).

### Regulation of FtsH Involved in Photosystem II Repair

Several proteins associated with the thylakoid membranes are reported to regulate FtsH activity. Psb29 from *Synechocystis* plays an important role in the accumulation of FtsH. Psb29 is a “pin” shaped protein that is needed for formation of the FtsH2/FtsH3 complex (Bečková et al., 2017). *Synechocystis* mutants lacking Psb29 are more susceptible to light stress, although not as severe as that of the FtsH2 deletion mutant. Psb29 interacts with FtsH2/FtsH3 complexes *in vivo*, however, the regulatory mechanism requires further investigation. THF1, which is the ortholog of Psb29 in plants, appears to play an equivalent role as levels of FtsH in *Arabidopsis* mutants lacking THF1 are also repressed, and the plants exhibit defective thylakoid formation and leaf variegation (Wang et al., 2004; Huang et al., 2013).

EngA is a GTPase that directly interacts with FtsH complexes. Its abundance negatively correlates with FtsH activities (Kato et al., 2018) so that *Arabidopsis* mutants over-expressing EngA display a leaf-variegation phenotype that is comparable to the FtsH-deficient *var* mutants (Chen et al., 2000; Takechi et al., 2000). Thylakoid FtsH exhibits a high turnover rate, especially under strong light (Zaltsman et al., 2005a; Li et al., 2017). EngA is therefore postulated to be a negative regulator acting on FtsH turnover (Kato et al., 2018).

FIP is a small thylakoid-anchored protein carrying a zinc-finger domain at the C-terminus (Lopes et al., 2018). It interacts with type A FtsH subunits in *Arabidopsis*, and possibly plays a role in assembly. The expression of FIP is downregulated when plants are exposed to abiotic stress, including excessive light, salt, oxidative agents and osmotic pressure. FIP knockdown mutants also display greater resilience to stress conditions. Hence, FIP negatively regulates FtsH activity. Unlike EngA mutants, over-expression of FIP does not result in aberrant plant development (Wang et al., 2017). Therefore, the impact of FIP on FtsH could be weaker. Further proteomic analysis on FIP mutants would help to clarify the role of FIP, as the initial report largely focused on a transcriptional analysis (Lopes et al., 2018).

### The Potential Co-evolution of Photosystem II and FtsH

The FtsH-mediated PSII repair cycle is a conserved feature for efficient oxygenic photosynthesis (Shao et al., 2018). Thus, understanding the co-evolution of PSII and FtsH might provide insights into the origin of oxygenic photosynthesis. A recent phylogenetic analysis has revealed that the FtsH protease complex involved in PSII repair forms a separate clade and is independent of the clade of FtsH protease subunits found in extant anoxygenic photosynthetic bacteria (Shao et al., 2018). Hence, the evolution timeline of the FtsH protease involved in PSII repair seems to match the early origin of PSII (Shao et al., 2018). In addition, the branching of the phylogenetic tree for FtsH in phototrophic bacteria closely resembles that of the type I and type II reaction centers, supporting the gene duplication hypothesis of oxygenic photosynthesis instead of horizontal gene transfer (Shao et al., 2018).

## Respiration and FtsH

### FtsH Proteases Maintain Protein Quality in Mitochondria

Like chloroplasts, mitochondria are also organelles of prokaryotic origin that were acquired by a eukaryotic host via endosymbiosis (Osteryoung and Nunnari, 2003). Mitochondria are cellular powerhouses dedicated to the supply of cellular ATP through aerobic respiration (Bertram et al., 2006). The respiratory reactions, also known as oxidative phosphorylation (OXPHOS), take place in the inner membrane of mitochondria (Chaban et al., 2014). Respiratory complexes embedded in the mitochondrial inner membrane perform a series of redox reactions that lead to the reduction of molecular oxygen to water and the formation of a proton motive force across the membrane which is then used to produce ATP via the ATP synthase (Hatefi, 1985; Chaban et al., 2014). Respiratory complexes are also prone to oxidative stress and require maintenance (Remmen and Richardson, 2001; Sweetlove et al., 2002; Augustin et al., 2005).

**FIGURE 4:**
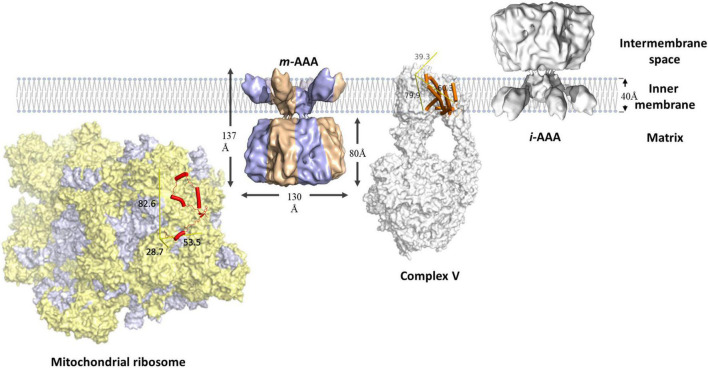
Schematic representation of mitochondrial FtsH complexes. The *i*-AAA is a homocomplex facing the intermembrane space, the *m*-AAA is a heterocomplex (blue and bronze) facing the mitochondrial matrix. Both complexes are modeled using the cryo-EM structure of yeast *m*-AAA (Lee et al., 2011). The unassembled F_*o*_a (orange cylinders) subunit of respiratory complex V (PDB: 6CP6) is a membrane protein substrate of *m*-AAA. MrpL32 (red cylinders) is a subunit of the mitochondrial ribosome (PDB: 5MRC), which consists of protein (yellow) and RNA (purple-gray) components. MrpL32 is a soluble substrate that requires post-translational modification by *m*-AAA. The proteins are scaled using the membrane width as reference. The yellow reference rulers represent the dimension (unit: Å) of the Inertia Axis Aligned Bounding Box (IAAB) for the respective protein, which are calculated by Guardado-Calvo’s Python scripts for pymol (https://pymolwiki.org/index.php/Draw_Protein_Dimensions).

FtsH proteases are found on both sides of the mitochondrial inner membrane, namely *m*-AAA and *i*-AAA, originally identified in studies on yeast (Thorsness et al., 1993; Guelin et al., 1994; Tzagoloff et al., 1994; Leonhard et al., 1996). The soluble domains of *m*-AAA are exposed to the inner matrix, whereas that of *i-*AAA face the intermembrane space (Figure 4; Leonhard et al., 1996; Steele and Glynn, 2019). Mitochondrial FtsH complexes target diverse substrates and exhibit functional overlap with other proteases. The maintenance of some respiratory complexes requires the participation of both *m*-AAA and *i*-AAA. For example, the stability of ATP synthase is reduced in *Arabidopsis* and yeast mutants lacking either *m*-AAA or *i*-AAA. However, *Arabidopsis* mutants lacking *i*-AAA retain the ability to degrade the unassembled *a* subunit of the ATP synthase (Marta et al., 2007). These results indicate that both FtsH complexes are involved in the assembly of ATP synthase, but *m*-AAA might be crucial for removal of damaged or unassembled ATP synthase subunits. Other respiratory proteins that require *m*-AAA-mediated maintenance include cytochrome *bc*_1_ and cytochrome *c* oxidase (Guélin et al., 1996; Stiburek et al., 2012). The *m*-AAA protease also plays a crucial role in ribosomal protein biosynthesis. It facilitates post-translational maturation of MrpL32, a subunit of the mitochondrial ribosome that is essential to assemble functional ribosomes (Nolden et al., 2005).

*i*-AAA on the opposite side plays a crucial role in maintaining lipid metabolism in mitochondria. Phosphatidylethanolamine and cardiolipin constitute up to 50% of the phospholipid in the inner membrane (Calzada et al., 2016; Schlame and Greenberg, 2017; Tatsuta and Langer, 2017). However, their biosynthesis requires the phosphatidylserine and phosphatidic acid precursors to be transported across the intermembrane space (Calzada et al., 2016; Schlame and Greenberg, 2017; Tatsuta and Langer, 2017). *i*-AAA regulates membrane biogenesis via restricting the number of lipid transfer proteins, e.g., PRELID1 and STARD7 (Potting et al., 2010; Wai et al., 2016; Saita et al., 2018). *i*-AAA also regulates protein transport between the cytoplasm and mitochondria by selective removal of the TIM translocase (Baker et al., 2012; Spiller et al., 2015). The release of cytochrome *c* from mitochondria during cell apoptosis is also regulated by *i*-AAA (Jiang et al., 2014; Saita et al., 2017).

### Functions and Regulation of Mitochondrial FtsH Complexes

Mitochondrial FtsH complexes are thought to have functional overlaps with other mitochondrial proteases, hence it is challenging to pinpoint the molecular targets of FtsH complexes (Patron et al., 2018). However, many links between FtsH mutations and diseases are well established through medical research (Karlberg et al., 2009; Gerdes et al., 2012; Patron et al., 2018). Human *i*-AAA mutations that destabilize complex formation lead to neuromuscular disorders including intellectual disability, motor developmental delay, optic atrophy, ataxia and movement deficiencies (Hartmann et al., 2016; Sprenger et al., 2019). Two types of *m*-AAA proteases are present in human: the AFG3L2 homocomplex and the AFG3L2/SPG7 heterocomplex (Martinelli et al., 2009; Tatsuta and Langer, 2009; Pierson et al., 2011). Recessive mutations in SPG7 (paraplegin) cause hereditary spastic paraplegia (HSP7) (Casari et al., 1998). The clinical features include weakness and spasticity of the lower limbs, loss of vibratory sense and urinary urgency (Patron et al., 2018). Mutations in AFG3L2 cause spinocerebellar ataxia type 28 (SCA28), a juvenile-onset disease featuring progressive gait and limb ataxia with abnormal eye movement (Cagnoli et al., 2006; Di Bella et al., 2010). A potential explanation for these FtsH-related diseases is that impaired FtsH activities destabilize respiration performance, leading to reduced energy output. The development and operation of the nervous system is particularly energy dependent, hence mitochondrial FtsH dysfunction causes severe neurodegenerative diseases (Tymianski and Tator, 1996; Adamo et al., 1999; Langer, 2000; Lee and Bendayan, 2004).

In plants, several mitochondrial FtsH subunits are crucial to mitigate heat stress. For example, *Arabidopsis* mutants lacking FtsH11 fail to grow at 30°C (Janska et al., 2010). A similar phenomenon is observed in the maize *ndl-1* mutants (Liu et al., 2019). A striking discrepancy is that a phylogenetic analysis suggests Ndl-1 is an *m*-AAA protease closely related to FtsH3 and FtsH10 in *Arabidopsis*. However, *Arabidopsis* mutants lacking FtsH3 or FtsH10 display a wild-type-like phenotype, and the mutants lacking both proteins only exhibit a minor growth defect, i.e., smaller size and shorter roots (Kolodziejczak et al., 2018; Liu et al., 2019). FtsH11 in *Arabidopsis* belongs to the *i*-AAA group, therefore, Ndl-1 and FtsH11 should be localized on the opposite side of the mitochondrial inner membrane. This apparent inconsistency in phenotype between the two plant species might reflect functional divergence of the FtsH proteases during evolution.

Prohibitins and stomatins are prominent modulators of the activities of mitochondrial FtsH proteases. As membrane scaffolding proteins, they act on the ultrastructure and dynamics of mitochondrial inner membranes (Artal-Sanz and Tavernarakis, 2009; Ikon and Ryan, 2017). Mutagenesis studies show that the activity of *m*-AAA is negatively correlated to the abundance of prohibitins (Koppen et al., 2007; Piechota et al., 2010). On the opposite side of the membrane, *i*-AAA complexes form super-complexes with stomatins (Wai et al., 2016). It is postulated that stomatins play a similar modulator role to prohibitins in regulating *i*-AAA activity in the intermembrane space (Wai et al., 2016). The molecular interactions between mitochondrial FtsH complexes and prohibitins or stomatins could be similar to that recently described for the interaction between *E. coli* HflK/C and FtsH (Yokoyama and Matsui, 2020; Ma et al., 2022).

## Evolution and Divergence of FtsH in Oxygenic Photosynthetic Organisms

### Photosynthetic Organisms Contain Diverse FtsH Complexes

**FIGURE 5:**
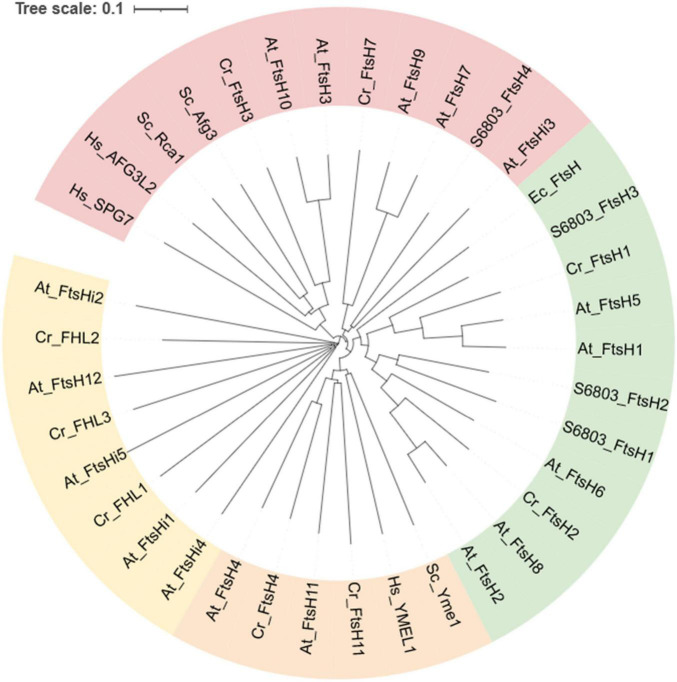
Phylogenetic analysis of FtsH orthologs from selected species. Annotations are: FtsH from *Escherichia coli* (begins with “Ec”), *Synechocystis* sp. PCC 6803 (begins with “S6803”), *Saccharomyces cerevisiae* S288C (begins with “Sc”), *Homo sapiens* (begins with “Hs”), *Chlamydomonas reinhardtii* (begins with “Cr”), and *Arabidopsis thaliana* (begins with “At”). Homologs with pale-green background correspond to Group1, that of pink correspond to Group2, orange and yellow correspond to Group3 as designated in the Shao et al. (2018) article. The tree was generated using MUSCLE (https://www.ebi.ac.uk/Tools/msa/muscle/). The sequences of At_FtsH were obtained from Tair (https://www.arabidopsis.org/index.jsp), and that of Cr_FtsH were collected from Phytozome *Chlamydomonas reinhardtii* v5.6 (https://phytozome-next.jgi.doe.gov). The remaining sequences were collected from NCBI (https://www.ncbi.nlm.nih.gov/protein/). The information was accessed on November 10, 2021.

Phylogeny mapping of 6,028 FtsH orthologs from 3,100 species has revealed that cyanobacteria carry ∼4 FtsH homologs per genome, the highest number among prokaryotes. Whereas the average number of FtsH homologs in fungal genomes is 2; that of animals is 4 and that of photosynthetic eukaryotes is 8 (Shao et al., 2018). This phylogenetic analysis concluded that FtsH orthologs cluster into three groups, Group 1 contains most bacterial and photosynthesis-related FtsH, Group 2 contains *m*-AAA forming homologs, Group 3 contains *i*-AAA-related FtsH and FtsHi (Figure 5). However, there are exceptions, e.g., FtsHi3 from *Arabidopsis* belongs to Group 2. Therefore, the FtsHi proteases in plants have a mixed ancestry.

### FtsH in Cyanobacteria

Cyanobacteria are a phylum of prokaryotes that perform oxygenic photosynthesis. It is widely accepted that chloroplasts in algae and plants are derived from an ancient cyanobacterium via endosymbiosis (Raven and Allen, 2003; Sakamoto et al., 2004; Kutschera and Niklas, 2005; Ayuso-Tejedor et al., 2010; Falcón et al., 2010). Like chloroplasts, cyanobacteria feature a thylakoid membrane system where photosynthesis takes place. Three classes of FtsH complexes are present in the *Synechocystis* model, the FtsH1/FtsH3 complex is present in cytoplasmic membranes and regulates iron acquisition (Boehm et al., 2012; Krynická et al., 2014, 2019); FtsH2/FtsH3 is anchored in the thylakoid membranes and is involved in PSII repair, osmoregulation and inorganic carbon assimilation (Stirnberg et al., 2007; Zhang et al., 2007; Boehm et al., 2012); the FtsH4 homocomplex is also located in the thylakoid membranes but its function is not yet clear (Silva et al., 2003; Boehm et al., 2012; Krynická et al., 2014; Sacharz et al., 2015).

In spite of different cellular localizations, FtsH1 and FtsH2 share the highest sequence identity and are both categorized as type B FtsH; while their partner FtsH3 resembles the type A FtsH subunits described for *Arabidopsis* (Sakamoto et al., 2003; Bečková et al., 2017). In both cyanobacteria and plants, disruption of type B FtsH leads to a dramatic reduction in levels of the thylakoid type A subunit (Sakamoto et al., 2003; Boehm et al., 2012). The speculation is that unassembled FtsH subunits are prone to degradation, hence unable to accumulate in the thylakoid membranes (Boehm et al., 2012). This hypothesis is further strengthened by the work on the FtsH1/FtsH3 complex, in which controlled repression of FtsH3 also led to a reduction in FtsH1 levels in *Synechocystis* (Krynická et al., 2014).

The physiological function of FtsH4 is not yet clear. Confocal microscopy of mutants expressing GFP-tagged FtsH4 revealed that FtsH4 is exclusively localized in the thylakoid membranes (Krynická et al., 2014; Sacharz et al., 2015). However, deletion of FtsH4 has not yet yielded a distinctive phenotype (Mann et al., 2000; Bailey et al., 2001). We speculate that FtsH4 could be a “failsafe” option for a broad range of degradants, which are predominantly removed via other proteases than the AAA + family of proteases. Hence probing the function of FtsH4 might require disruption of multiple proteases. Biochemical analysis shows that although the thylakoid membranes contain FtsH2, FtsH3, and FtsH4 subunits, the pairing of the protomers to produce hexameric complexes is strictly controlled by the FtsH type; no FtsH4 heterocomplexes have been reported.

### FtsH in Algae and Plants

Photosynthetic eukaryotes contain increased numbers of FtsH subunits. Green algae are the evolutionary link between cyanobacteria and plants, hence it is unsurprising that the number of FtsH homologs in algae is more than that of cyanobacteria and fewer than plants. For instance, the model green alga *C. reinhardtii* encodes six FtsH and three FtsHi homologs (Malnoë et al., 2014; Mishra and Funk, 2021; Zou and Bozhkov, 2021). FtsHi (recently reviewed by Mishra and Funk, 2021) represents a clade of FtsH with inactivated proteolytic capacity. Among the six homologs, CrFtsH1 and CrFtsH2 are involved in the quality control of thylakoid membrane proteins, such as the repair of PSII and the removal of cytochrome *b_6_f* (Malnoë et al., 2014; Bujaldon et al., 2017; Wang et al., 2017).

In *A. thaliana*, 12 FtsH and 5 FtsHi homologs have been identified (Wagner et al., 2012). They form five paired branches in the phylogram: AtFtsH1/5, AtFtsH2/8, AtFtsH7/9, AtFtsH3/10, and AtFtsH4/11 (Figure 5). The FtsH isoforms of each pair are functionally redundant and structurally interchangeable, except for the AtFtsH4/11 pair (Janska et al., 2010; Wagner et al., 2012; Mishra and Funk, 2021). Numerous studies have been conducted to elucidate the functions of each FtsH homolog (results summarized in Table 1).

**TABLE 1:** Summary of the phenotypes from FtsH mutants of *Arabidopsis thaliana*.

Genotype	FtsH location	Phenotype	Reference(s)
*ftsH1* (typeA) null	Thylakoid membrane	WT-like phenotype	Sakamoto et al., 2003; Zaltsman et al., 2005b
*ftsH5* (type A) null	Thylakoid membrane	Weak leaf variegation (var1)	Sakamoto et al., 2002; Wagner et al., 2011
*ftsH1*, *ftsH5* null	Thylakoid membrane	Albino-like, embryo lethality, loss of photoautotrophic growth	Yu et al., 2004, 2005; Zaltsman et al., 2005b
*ftsH2* (type B) null	Thylakoid membrane	Yellow variegated 2 (var2) severe leaf variegation	Chen et al., 2000; Takechi et al., 2000; Bailey et al., 2002; Wagner et al., 2011
*ftsH8* (type B) null	Thylakoid membrane	WT-like phenotype	Sakamoto et al., 2003; Zaltsman et al., 2005b; Wagner et al., 2011
*ftsH2*, *ftsH8* null	Thylakoid membrane	Albino-like, embryo lethality, loss of photoautotrophic growth	Yu et al., 2004, 2005; Zaltsman et al., 2005b
*ftsH1*, *ftsH8* null	Thylakoid membrane	WT-like phenotype	Zaltsman et al., 2005b
*ftsH6* null	Thylakoid membrane	Enhanced heat-tolerance and thermomemory	Sedaghatmehr et al., 2016
*ftsH7* null	Chloroplast envelope	WT-like phenotype	Wagner et al., 2011
*ftsH9* null	Chloroplast envelope	Unknown	Ferro et al., 2010; Wagner et al., 2011
*ftsH12* null	Chloroplast envelope	Embryo lethality	Ferro et al., 2010
*ftsHi1* null	Chloroplast envelope	Embryo lethality	Kadirjan-Kalbach et al., 2012; Mishra et al., 2019
*ftsHi1* missense mutant	Chloroplast envelope	Pale seedling	Kadirjan-Kalbach et al., 2012; Mishra et al., 2019
*ftsHi2* null	Chloroplast envelope	Embryo lethality	Schreier et al., 2018; Mishra et al., 2019
*ftsHi3* null	Chloroplast envelope	Residual albino growth	Kikuchi et al., 2018
*ftsHi4* null	Chloroplast envelope or thylakoid membrane	Embryo lethality	Lu et al., 2014; Mishra et al., 2019
*ftsHi5* null	Chloroplast envelope	Embryo lethality	Wang et al., 2018; Mishra et al., 2019
*ftsH11* (*i*-AAA) null	Chloroplast envelope	Reduced heat-tolerance	Urantowka et al., 2005; Chen et al., 2006; Wagner et al., 2011, 2016
*ftsH4* (*i*-AAA) null	Mitochondrial inner membrane	Abnormal leaf morphology in late rosette development under short-day conditions	Gibala et al., 2009; Wagner et al., 2011
*ftsH3* (*m*-AAA) null	Mitochondrial inner membrane	WT-like phenotype	Piechota et al., 2010; Wagner et al., 2011
*ftsH10* (*m*-AAA) null	Mitochondrial inner membrane	WT-like phenotype	Piechota et al., 2010; Wagner et al., 2011
*ftsH3*, *ftsH10* null	Mitochondrial inner membrane	Decreased size of seedlings and developmental delay	Kolodziejczak et al., 2018

Five FtsH homologs are localized on the thylakoid membrane. AtFtsH1 and 5 belong to type A FtsH and AtFtsH2 and 8 are type B (Wagner et al., 2012). Together, they form heterocomplexes responsible for PSII repair (Kato and Sakamoto, 2018). AtFtsH2 and AtFtsH5 are the dominant isoforms in thylakoid membranes; disruption of either homolog leads to leaf-variegation phenotypes (Chen et al., 2000; Takechi et al., 2000; Sakamoto et al., 2002; Zaltsman et al., 2005b). Mutants lacking AtFtsH1 and AtFtsH8 do not display a clear change in phenotype to WT, however, dual disruption of AtFtsH1/5 or AtFtsH2/8 produces mutants with severe albino-like leaves. Hence, AtFtsH1 and 8 contribute to activity, but are insufficient to substitute for the dominant isoform (Wagner et al., 2012). Over-expression of AtFtsH8 can complement the loss of functional AtFtsH2, indicating the physiological impact of AtFtsH8 is restricted by its quantity in the wild-type plants (Yu et al., 2004).

The import of FtsH into the chloroplast and insertion into the thylakoid membrane is determined by the N-terminal sequence. Type A FtsH subunits are inserted via the secretion pathway (Sec), which inserts unfolded proteins into the membrane; however, type B FtsH subunits are inserted via the twin-arginine translocation pathway (TAT), which transports fully folded protein across the membrane (Rodrigues et al., 2011). *In vitro* import studies suggest that the mature thylakoid FtsH subunits from *Arabidopsis* might contain a single transmembrane alpha helix (Rodrigues et al., 2011) rather than the two transmembrane helices predicted for cyanobacterial FtsH subunits based on N-terminal sequencing of the protein subunits (Boehm et al., 2012).

The role of thylakoid AtFtsH6 remain elusive, despite its wide conservation in plants (Sun et al., 2006; Johnson et al., 2014; Yu et al., 2014; Xue et al., 2015). AtFtsH6 is rapidly induced under heat stress, and is involved in the degradation of heat-shock protein HSP21 (Sedaghatmehr et al., 2016). Mutants lacking AtFtsH6 are reported to display stronger thermomemory and thermotolerance, but the phenotype is weak (Sedaghatmehr et al., 2016).

In *Arabidopsis*, the following FtsH isoforms are anchored in the chloroplast envelope membrane: FtsH7, 9, 12 and FtsHi1-i5 (Kadirjan-Kalbach et al., 2012; Wagner et al., 2012; Mishra et al., 2019). Notably, FtsH7, 9 and i3 are members of the Group 2 clade (Figure 5). Both FtsH7 and FtsH9 contain an additional protease domain between the second transmembrane helix and the ATPase domain. FtsHi3 also contains a protease domain upstream of the ATPase domain, however, its zinc-binding catalytic site is missing, and the C-terminal protease domain is also lost (Mishra and Funk, 2021). Mutants lacking AtFtsH7 retain a wild-type-like phenotype and the function of AtFtsH9 is unclear (Wagner et al., 2011). In contrast, the remaining FtsH homologs found in the chloroplast envelope membrane are essential for embryogenesis (Wagner et al., 2012; Mishra and Funk, 2021).

In *Arabidopsis*, FtsHi1, 2, 4, and 5 lack the HEXXH motif necessary for substrate degradation (Figure 1); whereas the entire protease-inactive domain of FtsHi3 is relocated upstream of the ATPase domain (Wagner et al., 2012; Mishra and Funk, 2021). FtsH12 and FtsHi have been speculated to form complexes, because the mortality rate of a FtsH12 knockout mutant is comparable to that of FtsHi2, 4, and 5 single mutants (Ferro et al., 2010; Wagner et al., 2012). Four FtsHi (FtsHi1, 2, 4, and 5) have been confirmed to form complexes with Ycf2 and AtFtsH12 on the stroma-side of the chloroplast envelope and are thought to be involved in substrate transport (Kikuchi et al., 2018). Interestingly, Ycf2 is a distant relative of FtsH that has remained encoded by the chloroplast genome (Downie et al., 1994; Bungard, 2004). A phylogenetic analysis of Ycf2 suggests the gradual loss of the protease catalytic site during evolution, and the enlargement of the soluble region between the two transmembrane helices (Kikuchi et al., 2018).

AtFtsH3/4/10 are found in mitochondria. AtFtsH3 and AtFtsH10 are *m*-AAA proteases that form both homo- and heterocomplexes (Janska et al., 2010). Surprisingly, neither of them is essential for growth under optimal conditions (Wagner et al., 2012). AtFtsH4 and AtFtsH11 are related to *i-*AAA, and are present as homocomplexes in mitochondria (Janska et al., 2010). AtFtsH4-deficient mutants develop a distinct asymmetric shape and irregular serration of expanding leaf blades (Gibala et al., 2009). Despite being paired with AtFtsH4, AtFtsH11 was thought to be targeted to both mitochondria and chloroplasts (Urantowka et al., 2005), however, recent work suggests that AtFtsH11 is exclusively targeted to the chloroplast envelope (Ferro et al., 2010; Wagner et al., 2016). Mutants lacking AtFtsH11 are sensitive to high temperature with growth arrested at 30°C (Janska et al., 2010).

In summary, FtsH homologs in plants preserve genetic redundancy, functional overlap, and structural plasticity to deliver diverse functions. The functional redundancy among isoforms enables FtsH homologs to adapt to emerging environmental demands, resulting in a robust and flexible system to maintain cellular homeostasis through evolution.

## Outlook

The FtsH family of proteases are versatile tools to regulate diverse pathways by removing both soluble and membrane-bound proteins (Kihara et al., 1995; Tomoyasu et al., 1995; Silva et al., 2003; Krynická et al., 2014). FtsH is proficient in maintaining membrane protein quality, hence plays a crucial role in energy metabolism, including photosynthesis and respiration. FtsH functions as a hexameric complex, with the type of protomer defining substrate specificity. The proteolytic activities can be further adjusted by modulator proteins. The molecular mechanisms that regulate FtsH activities at multiple levels make the FtsH-mediated proteolysis system reliable and adaptable.

The cellular localization and subunit composition of many FtsH complexes have been determined. However, we think the following questions could be important to address in the future. The first question is how the composition of FtsH complexes is controlled. Mutagenesis studies have revealed that the protease domain is important to prevent formation of undesirable protomer pairing, however, the protease domain is also variable across species. The high-resolution structures of intact FtsH complexes have not yet been determined but will be crucial for understanding how specific protomers are selected for assembly into heterocomplexes.

The second question is how substrates are targeted for degradation. Although a structure of a FtsH complex has been determined with a target protein stuck in the proteolytic chamber (Puchades et al., 2017), there is still no structural information regarding the entry of substrates through the central pore of the ATPase domain.

The third question is how the proteolytic activity of FtsH complexes is regulated. Despite recent advances in the structural analysis of the HflK/C-FtsH super-complex (Ma et al., 2022), the molecular mechanisms that modulate FtsH activities in the thylakoid and mitochondrial inner membrane remain unclear. In particular, further investigations are required to verify the role of prohibitins in photosynthetic and eukaryotic organisms.

## Author Contributions

LY participated in drafting the manuscript and offered first authorship. BL helped with project management and data analysis, offered second authorship. PN was an expert in this field, and gave guidance and support and helped draft the manuscript. JY supervised LY, worked together to craft the manuscript. FC was the team leader that brought all resources together for this project. PN, JY, and FC agreed to share correspondence. All authors contributed to the article and approved the submitted version.

## Conflict of Interest

The authors declare that the research was conducted in the absence of any commercial or financial relationships that could be construed as a potential conflict of interest.

## Publisher’s Note

All claims expressed in this article are solely those of the authors and do not necessarily represent those of their affiliated organizations, or those of the publisher, the editors and the reviewers. Any product that may be evaluated in this article, or claim that may be made by its manufacturer, is not guaranteed or endorsed by the publisher.
